# Arthroscopic Noninvasive Transtendon Double‐Pulley Suture‐Bridge Repair of Both‐Sided Partial‐Thickness Rotator Cuff Tears

**DOI:** 10.1002/atn2.70205

**Published:** 2026-07-31

**Authors:** Peiguan Huang, Hairong Tang, Xiaoxu Wang, Yong Fu, Zhengmao Li, Chunrong He

**Affiliations:** ^1^ The Second Affiliated Hospital Department of Joint Surgery Hengyang Medical School University of South China Hengyang China; ^2^ Hunan University of Medicine Huaihua China

## Abstract

Currently, surgical techniques for both‐sided partial‐thickness rotator cuff tears (PTRCTs) are rarely described, and the successful repair of both‐sided PTRCTs is challenging. Transtendon repair is a good technique for repairing PTRCTs, but the resulting trauma to the tendon is unacceptable. Therefore, we describe a method of noninvasive transtendon repair that does not require a medial‐row anchor. In the treatment of PTRCTs, the residual tendon can be thoroughly preserved without any trauma; a tendon tear on the bursa can be effectively repaired, while the articular layer can also be sufficiently restored. Six sets of double‐pulley suture‐bridge repair can offer superior failure strength and enough initial stability of the tendon.

## Graphical abstract

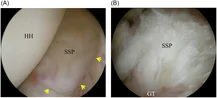

**VIDEO 1:** The patient is placed in the lateral decubitus position and undergoes a surgical procedure on the left shoulder under general anesthesia. A 1.7 × 0.7 cm tendon tear on the articular layer and a 3.2 × 2.5 cm tendon tear on the bursal layer are confirmed. The articular and bursal layer tears are not connected between the subacromial space and the glenohumeral joint. In total, 6 strands of 3 sutures, which are used as medial‐row sutures, are passed through the supraspinatus tendon and percutaneously introduced into the subacromial space, alternating between blue and white. Two suture anchors with blue and white strands are used as lateral‐row anchors. The blue strands from anchor A and white strands of suture 3 are incorporated into the first and second sets of DPSBs. The white strands from anchor B and the blue strands of suture 1 are incorporated into the third and fourth sets of DPSBs. The blue strands from anchor A and white strands of suture 2 are incorporated into the fifth and sixth sets of DPSBs. A total of 6 sets of DPSBs strongly press the supraspinatus tendon against the footprint. The torn portions of the bursal layer and articular layer are anatomically reduced with enough tendon tension. (DPSB, double‐pulley suture bridge.) Video content can be viewed at https://doi.org/10.1002/atn2.70205.

Both‐sided partial‐thickness rotator cuff tears (PTRCTs) comprise articular and bursal layer tears without a tear in the connection between the subacromial space and glenohumeral joint. A low incidence rate of both‐sided PTRCTs is generally expected but not uncommon.^1^ Radiological research shows a 20% incidence rate of both‐sided PTRCTs.^2^ However, surgical techniques for both‐sided PTRCTs are rarely described.^3^ The successful repair of both‐sided PTRCTs is challenging.^4^

Transtendon repair is a good method of treating both‐sided PTRCTs; the residual tissues of the tendon can be preserved, and a minimized length‐tension mismatch of the tendon can be obtained.^5^ Even so, the anchor piercing directly through the residual tendon is an apparent defect in transtendon repair;^6^ the tendon continues to degenerate due to the anchor piercing.^7^ Slow recovery of function and shoulder stiffness in patients having undergone transtendon repair have been shown.^8^, ^9^

Therefore, we describe a noninvasive transtendon repair technique that does not use a medial‐row anchor. In the treatment of both‐sided PTRCTs, all the residual tendon tissues can be thoroughly preserved; a tendon tear on the bursa can be effectively repaired, while the articular layer can also be sufficiently restored. Six sets of double‐pulley suture‐bridge (DPSB) repair can obtain enough initial fixation strength and tendon‐to‐bone coverage area.

## SURGICAL TECHNIQUE

### Patient Positioning and Anesthesia

The patient is placed in the lateral decubitus position and undergoes a surgical procedure on the left shoulder under general anesthesia (Video 1). Clinical pearls, pitfalls, advantages, and disadvantages are described in Tables 1 and 2. The study was approved by the ethical department, and the patient provided informed consent.

**TABLE 1:** Surgical Pearls and Pitfalls

Pearls
Medial‐row suture considerations
1. No. 2 nonabsorbable polyester suture is applied as medial‐row suture; medial‐row anchor is not used
2. The distance between 2 strands of suture 1 should be 6 to 8 mm
3. A total of 6 strands of 3 sutures are passed through the supraspinatus tendon and percutaneously introduced into the subacromial space, alternating between blue and white
Anchor insertion consideration
Two suture anchors are used as lateral‐row anchors and implanted in the greater tuberosity far away from the margins of the supraspinatus tendon
DPSB creation considerations
The blue strands from anchor A and white strands of suture 3 are incorporated into the first and second sets of DPSBs. The white strands from anchor B and the blue strands of suture 1 are incorporated into the third and fourth sets of DPSBs. The blue strands from anchor A and white strands of suture 2 are incorporated into the fifth and sixth sets of DPSBs

Pitfalls
1. A tear size on the articular layer of the tendon of more than 1.5 cm is not suitable for this technique; 4 sets of medial‐row sutures are needed
2. The knots of the DPSB should be securely tied to ensure the knot cannot be undone or loosened
3. When the suture is locked in the double‐pulley process, both sides of the 2 strands of the medial‐row suture and the lateral‐row anchor will be knotted with a knot pusher in the subacromial space

DPSB, double‐pulley suture bridge.

**TABLE 2:** Advantages and Disadvantages

Advantages
1. Because a medial‐row anchor is not used, there is no risk of the anchor puncturing through the tendon, and the residual tendon can be thoroughly preserved without any trauma
2. A tendon tear on the bursa can be effectively restored while the articular layer can also be sufficiently restored
3. Six sets of DPSBs can offer superior failure strength, enough initial stability of the tendon, and decreased gap formation
4. Both the time and difficulty of the surgical procedure can be lowered, because a medial‐row anchor is not required and suture‐relay procedures are simplified

Disadvantages
1. It is difficult to achieve anatomical reduction of an articular‐sided tendon tear because a medial‐row anchor is not used
2. The spinal needle punctures the shoulder skin multiple times, which could potentially lead to joint infection
3. This surgical technique demands high technical expertise of surgeons, and the learning curve is long

DPSB, double‐pulley suture bridge.

### Diagnostic Procedure

**FIGURE 1:**
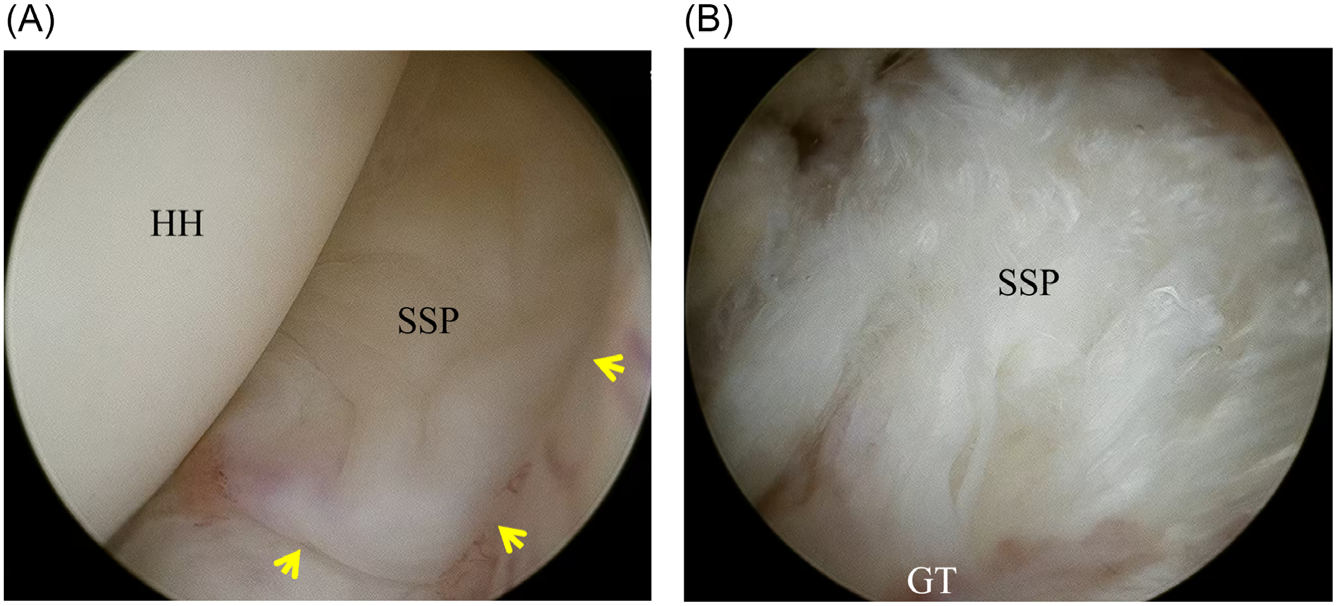
(A) Arthroscopic image of the left shoulder (lateral decubitus position) viewed through the posterior portal showing an Ellman grade 2 partial articular‐sided supraspinatus tendon avulsion lesion and 1.7 × 0.7 cm tendon tear. Yellow arrows: tear site of the tendon. (B) Arthroscopic image of the left shoulder (lateral decubitus position) viewed through the subacromial lateral portal showing that the tissue of the bursal layer is widely degenerated with longitudinal tears; the tendon attached to the GT is intact. (GT, greater tuberosity; HH, humeral head; SSP, supraspinatus tendon.)

Intra‐articular examination is performed from the posterior portal; the thickness and size of the supraspinatus tendon tear are assessed. An Ellman grade 2 PASTA lesion and a 1.7 × 0.7 cm tendon tear are confirmed (Figure 1A). The rotator interval is thoroughly cleared for an unhindered channel of suture relay. A tenotomy is performed on a SLAP lesion.

Then, the scope is introduced into the subacromial space, a bursectomy is performed, and an acromioplasty is performed on a hook acromion. The tissues of the bursal layer are widely degenerated with a longitudinal tear; the tendon attached to the greater tuberosity is intact (Figure 1B). Finally, a 1.7 × 0.7 cm tendon tear on the articular layer and a 3.2 × 2.5 cm tendon tear on the bursal layer are confirmed. The articular and bursal layer tears are not connected between the subacromial space and the glenohumeral joint.

### Medial‐Row Suture Process

**FIGURE 2:**
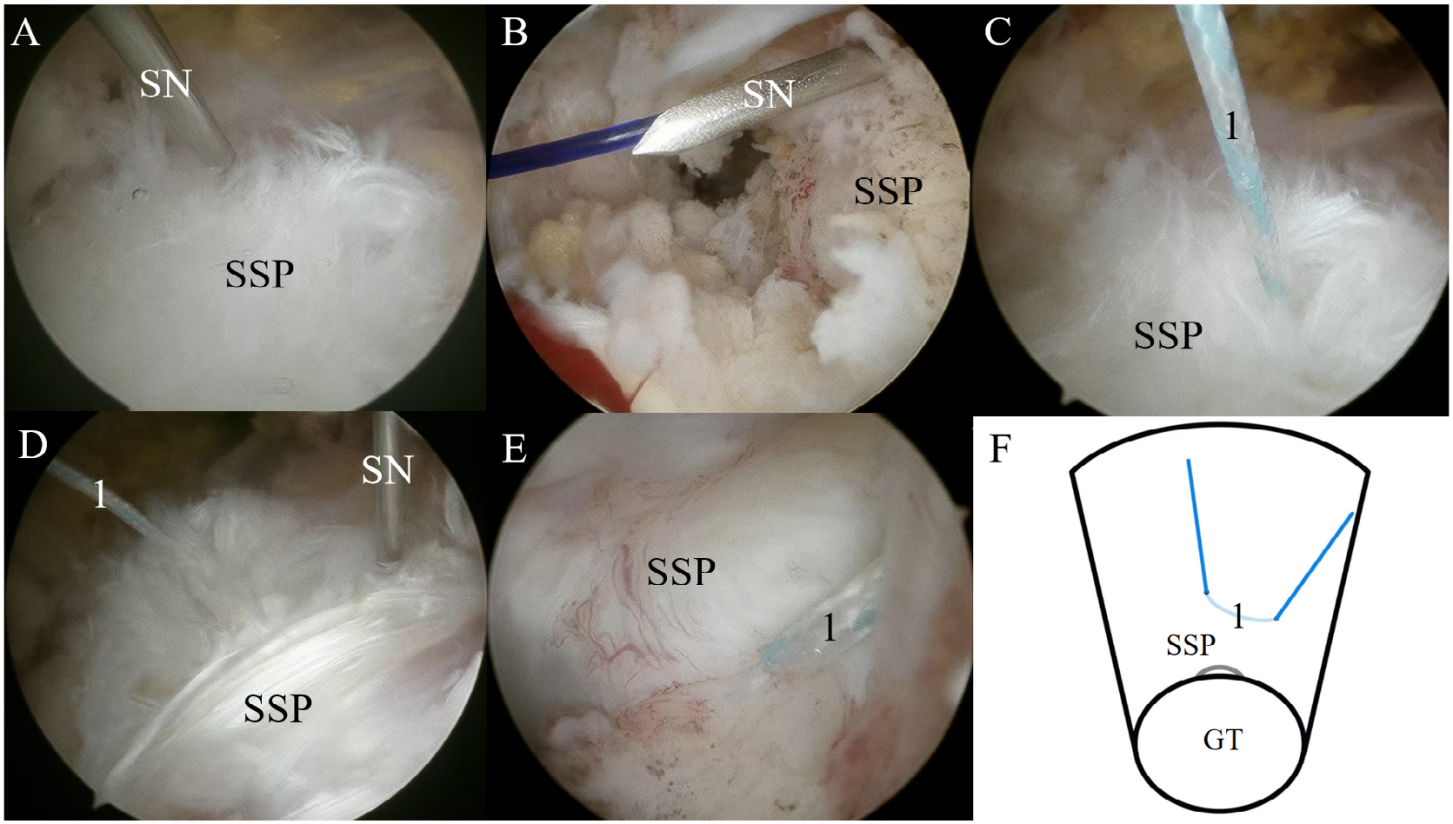
(A) Arthroscopic image of the left shoulder (lateral decubitus position) viewed through the subacromial lateral portal showing an 18‐gauge SN with a polydioxanone suture percutaneously pierced from the bursal layer into the articular cable. (B) Arthroscopic image of the left shoulder (lateral decubitus position) viewed through the posterior portal showing an 18‐gauge SN with a polydioxanone suture percutaneously pierced from the bursal layer into the articular cable. (C) Arthroscopic image of the left shoulder (lateral decubitus position) viewed through the subacromial lateral portal showing that the strand of suture 1 (1) is passed through the SSP and percutaneously introduced into the subacromial space. (D) Arthroscopic image of the left shoulder (lateral decubitus position) viewed through the subacromial lateral portal showing an SN with another PDS suture percutaneously pierced from the bursal layer to the articular cable again. (E) Arthroscopic image of the left shoulder (lateral decubitus position) viewed through the posterior portal showing that 2 strands of suture 1 (1) are passed through the SSP. (F) Illustration of the left shoulder showing that 2 strands of suture 1 (1) are percutaneously introduced into the subacromial space. (GT, greater tuberosity; PDS, polydioxanone; SN, spinal needle; SSP, supraspinatus tendon.)

In the subacromial space, an 18‐gauge spinal needle with a polydioxanone (PDS) suture is percutaneously pierced from the bursal layer (Figure 2A) into the articular cable (Figure 2B). In the joint, the PDS suture is passed through the rotator interval and removed from the body with forceps. A blue No. 2 nonabsorbable polyester suture (HealFix, REJOIN, Hangzhou, Zhejiang) is applied for the medial‐row suture and named suture 1. One strand of suture 1 is securely tied on the PDS suture in an extracorporeal manner. This strand of suture 1 will be passed through the supraspinatus tendon and percutaneously introduced into the subacromial space after pulling the opposite strand of PDS suture (Figure 2C).

A spinal needle with another PDS suture is percutaneously pierced from the bursal layer to the articular cable again (Figure 2D). The opposite strand of suture 1 is placed in the joint, and the PDS suture is removed from the body and securely tied. The opposite strand of suture 1 will also be percutaneously introduced into the subacromial space by pulling the opposite strand of PDS suture. The distance between 2 strands of suture 1 should be 6 to 8 mm (Figure 2E,F).

**FIGURE 3:**
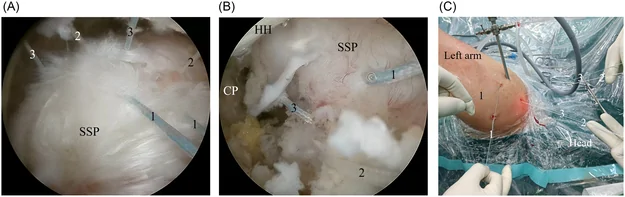
(A) Arthroscopic image of the left shoulder (lateral decubitus position) viewed through the subacromial lateral portal showing that a total of 6 strands of 3 sutures are passed through the SSP and percutaneously introduced into the subacromial space, alternating between blue and white. 1: the strands of suture 1; 2: the strands of suture 2; 3: the strands of suture 3. (B) Arthroscopic image of the left shoulder (lateral decubitus position) viewed through the posterior portal showing that a total of 6 strands of 3 sutures are passed through the SSP. 1: the strands of suture 1; 2: the strands of suture 2; 3: the strands of suture 3. (C) Intraoperative photo from a superior view showing a total of 6 strands from suture 1 (1), suture 2 (2), and suture 3 (3). (CP, coracoid process; HH, humeral head; SSP, supraspinatus tendon.)

A white suture named suture 2 and a new blue suture named suture 3 are placed using the same procedures as described previously. In total, 6 strands of 3 sutures are passed through the supraspinatus tendon and percutaneously introduced into the subacromial space, alternating between blue and white (Figure 3A‐C).

### Lateral‐Row Anchor Implantation

**FIGURE 4:**
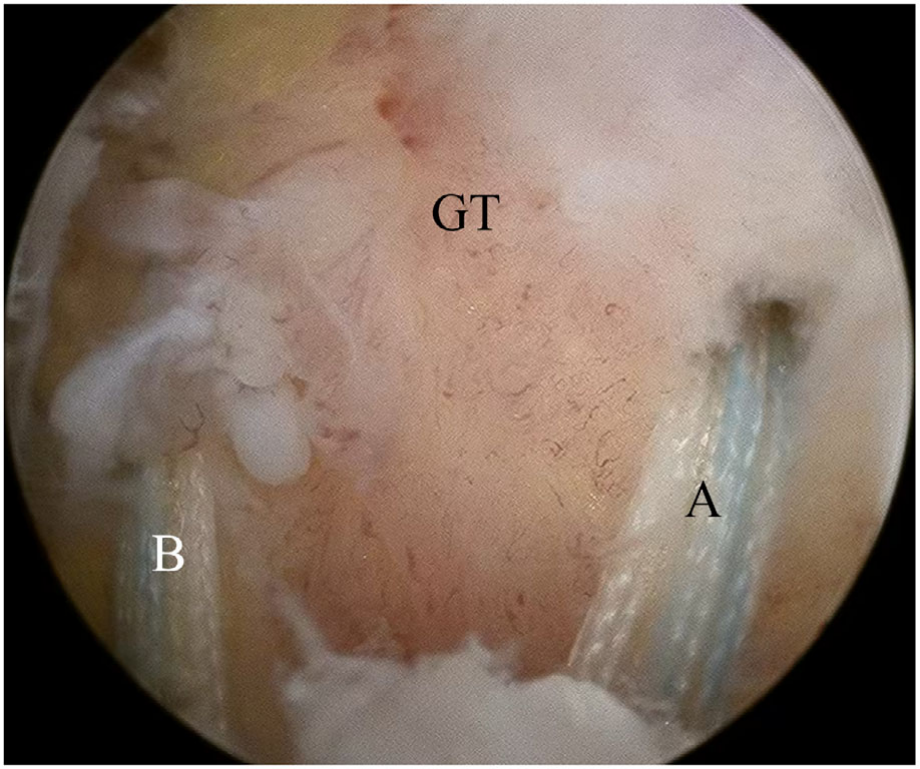
Arthroscopic image of left shoulder (lateral decubitus position) viewed through the subacromial lateral portal showing that 2 suture anchors with blue and white strands are implanted in the GT far away from the margins of the supraspinatus tendon. A: the sutures from anchor A; B: the sutures from anchor B. (GT, greater tuberosity.)

In the subacromial space, 2 suture anchors with blue and white strands (HealFix, REJOIN, Hangzhou, Zhejiang), named anchors A and B, are used as lateral‐row anchors. The 2 anchors are implanted in the greater tuberosity far away from the margin of the supraspinatus tendon (Figure 4).

### First DPSB Process

**FIGURE 5:**
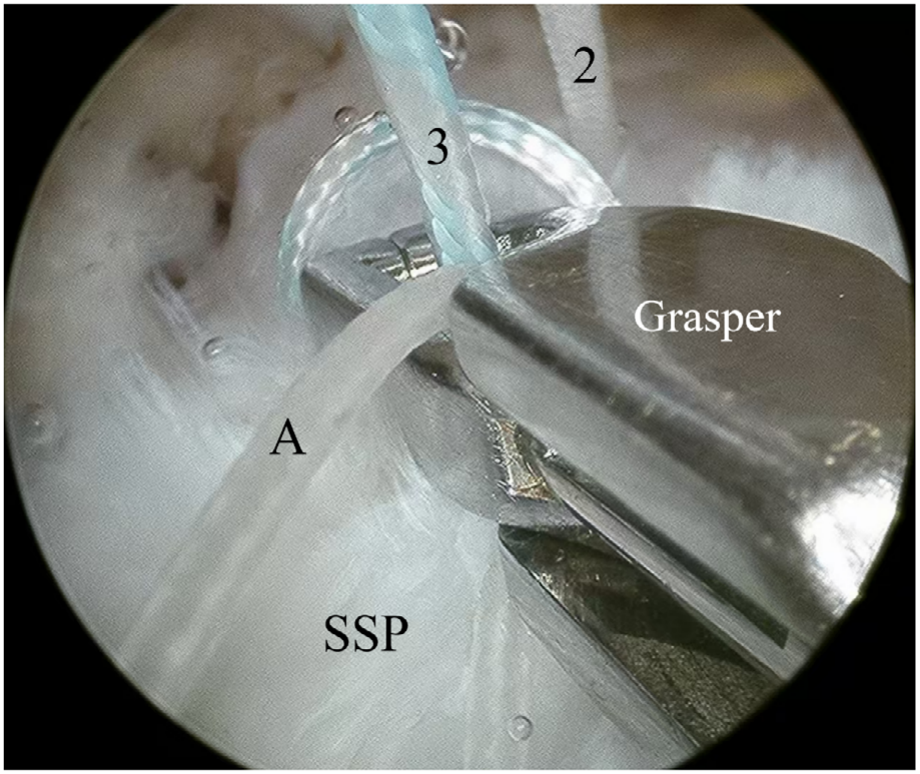
Arthroscopic image of the left shoulder (lateral decubitus position) viewed through the subacromial lateral portal showing that 1 white strand from anchor A (A) and 1 blue strand of suture 3 (3) are passed through the anterior part of the tendon and will be removed from the body. 2: the strands of suture 2. (SSP, supraspinatus tendon.)

**FIGURE 6:**
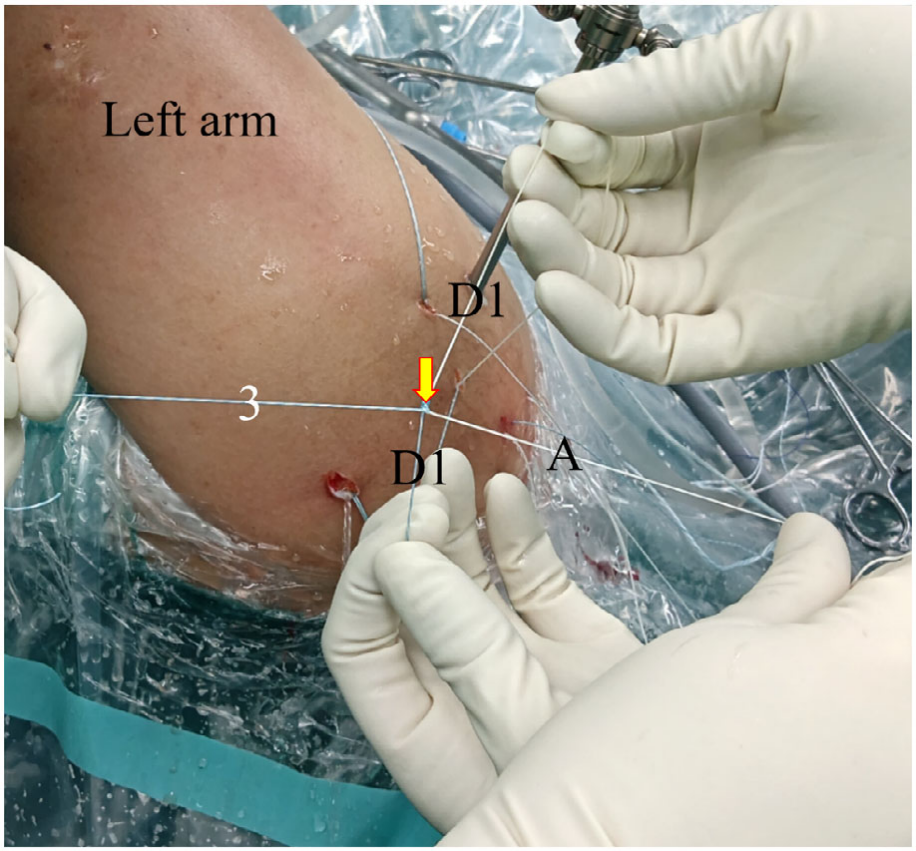
Intraoperative photo from a superior view showing that 1 white strand from anchor A (A) and 1 blue strand of suture 3 (3) are passed through the anterior part of the tendon and form the first set of double‐pulley suture bridges (D1).

In the subacromial space, 1 white strand from anchor A and 1 blue strand of suture 3 that passed through the anterior part of the tendon are removed from the body (Figure 5) and securely tied. This knotted blue‐white suture is regarded as the first set of DPSBs (Figure 6).

**FIGURE 7:**
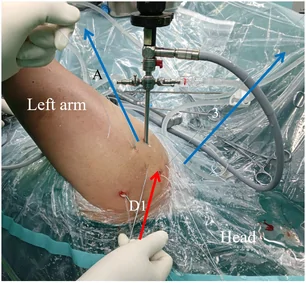
Intraoperative photo from a superior view showing that when the opposite strands of suture 3 and anchor A obtain sufficient tension, this set of DPSBs can strongly press the tendon against the footprint. 3: the strands of suture 3; A: the sutures from anchor A; D1: the first set of DPSB; blue arrow: the direction in which the strands are pulled out; red arrow: the direction in which the first set of DPSBs is drawn into the body. (DPSB, double‐pulley suture bridge.)

**FIGURE 8:**
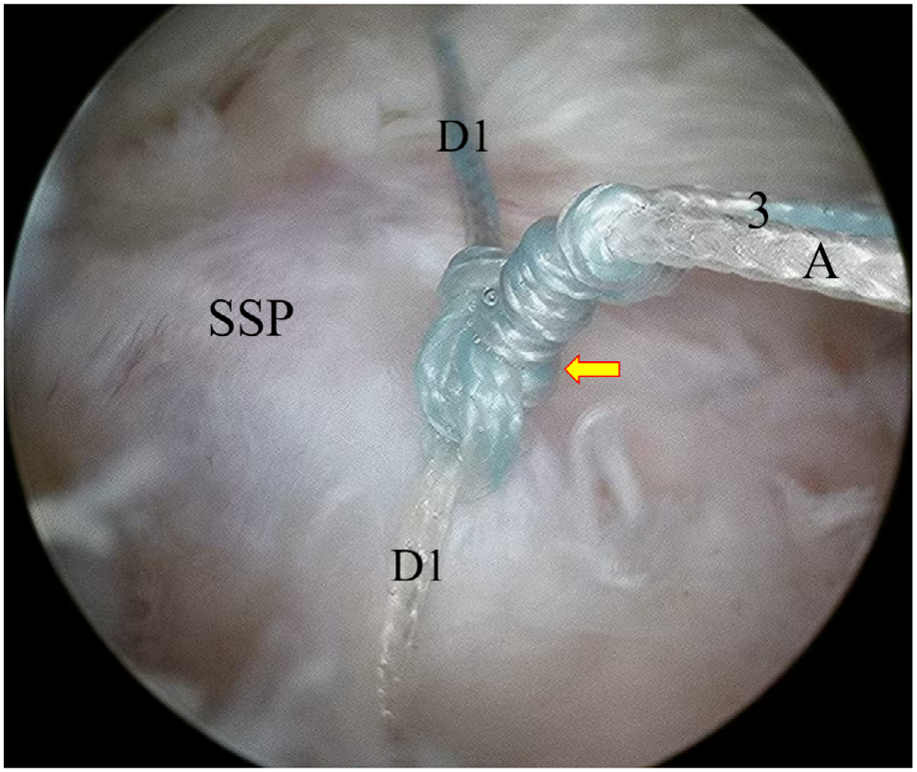
Arthroscopic image of the left shoulder (lateral decubitus position) viewed through the subacromial lateral portal showing that the first set of double‐pulley suture bridges (D1) can strongly press the tendon against the footprint. 3: the strands of suture 3; A: the sutures from anchor A. (SSP, supraspinatus tendon.)

Using a double‐pulley technique, this set of DPSBs along with the knot is gradually dragged into the subacromial space by pulling on the opposite strands of suture 3 and anchor A (Figure 7), and the suture strands above the knot are cut. When the opposite strands of suture 3 and anchor A obtain sufficient tension, this set of DPSBs can strongly press the tendon against the footprint (Figure 8).

### Second to Sixth DPSB Processes

**FIGURE 9:**
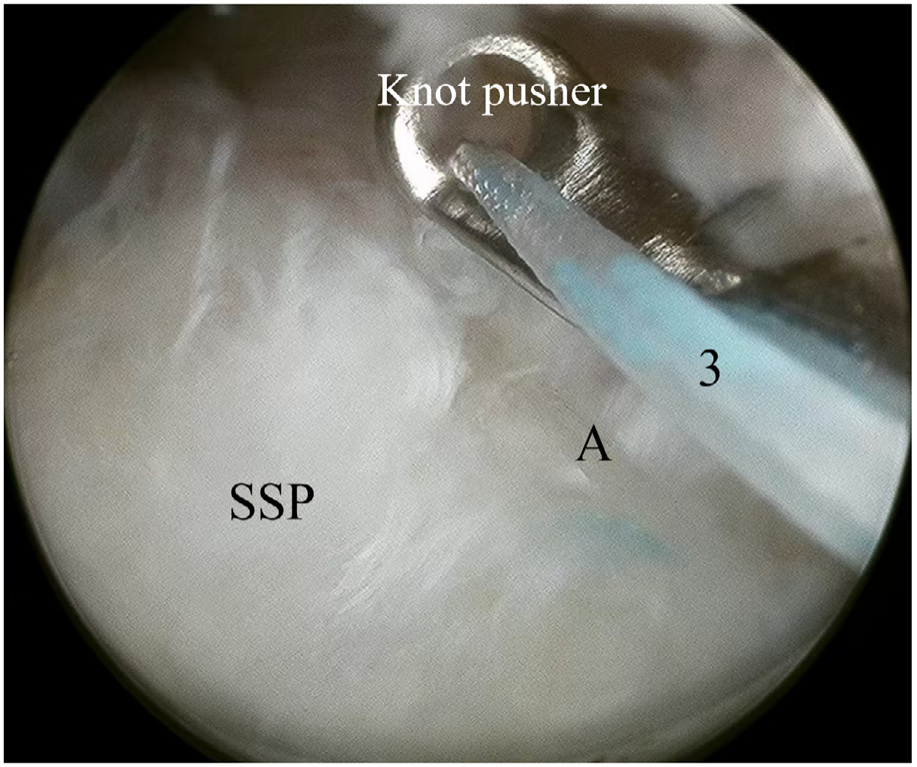
Arthroscopic image of the left shoulder (lateral decubitus position) viewed through the subacromial lateral portal showing that the opposite 2 strands of suture 3 and anchor A are removed from the body and securely tied using a knot pusher. 3: the strands of suture 3; A: the sutures from anchor A. (SSP, supraspinatus tendon.)

In the subacromial space, the opposite 2 strands of suture 3 and anchor A are removed from the body and securely tied by a knot pusher (Figure 9); the suture strands above the knot are also cut. This is the second set of DPSBs.

The white strands from anchor B and the blue strands of suture 1 are incorporated into the third and fourth sets of DPSBs. The blue strands from anchor A and white strands of suture 2 are incorporated into the fifth and sixth sets of DPSBs.

### Repaired Tendon Assessment

**FIGURE 10:**
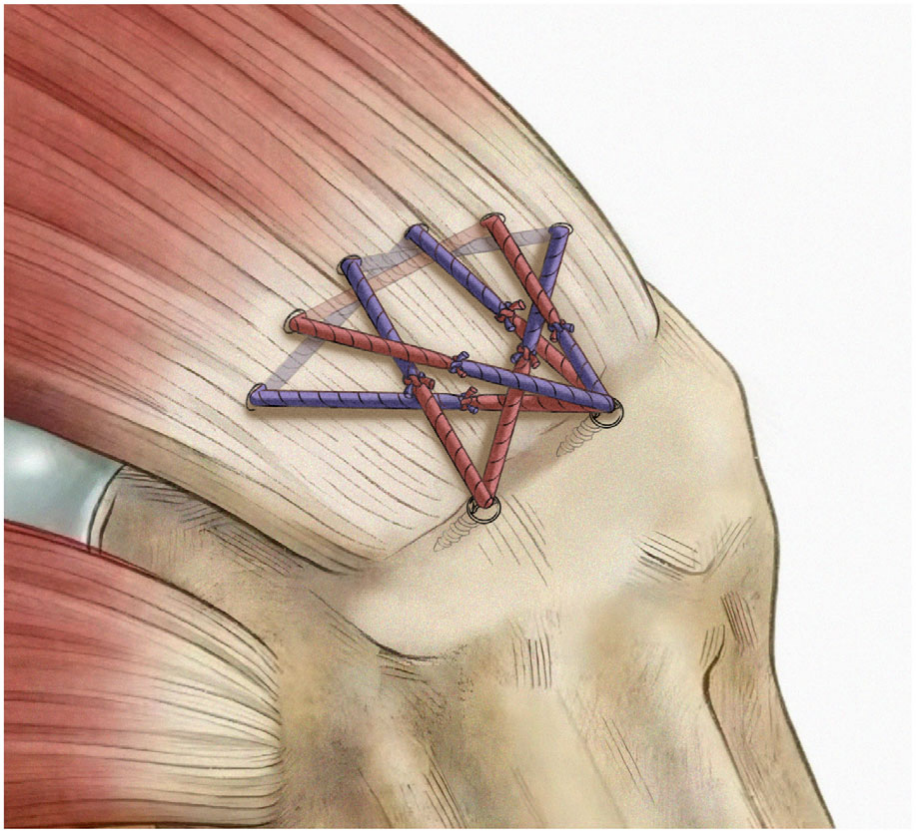
Illustration of the left shoulder showing that a total of 6 sets of double‐pulley suture bridges strongly press the supraspinatus tendon against the footprint.

**FIGURE 11:**
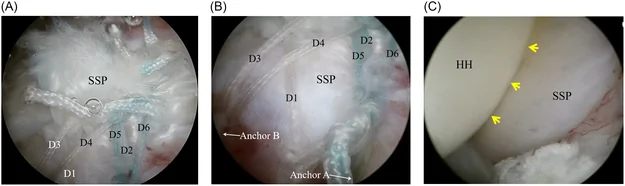
(A) Arthroscopic image of the left shoulder (lateral decubitus position) viewed through the subacromial lateral portal showing that the torn portion of the bursal layer is anatomically reduced with enough tendon tension. D1‐D6: the first to sixth sets of double‐pulley suture bridges. (B) Arthroscopic image of the left shoulder (lateral decubitus position) viewed through the subacromial lateral portal showing that the torn portion of the bursal layer is anatomically reduced with enough tendon tension. D1‐D6: the first to sixth sets of double‐pulley suture bridges; anchor A: the position of anchor A; anchor B: the position of anchor B. (C) Arthroscopic image of the left shoulder (lateral decubitus position) viewed through the posterior portal showing that the torn portion of the articular layer is anatomically reduced with enough tendon tension. Yellow arrows: the reduced tendon. (HH, humeral head; SSP, supraspinatus tendon.)

In the subacromial space, a total of 6 sets of DPSBs strongly press the supraspinatus tendon against the footprint (Figure 10). The torn portion of the bursal layer is anatomically reduced with enough tendon tension (Figure 11A,B). In the joint, the torn portion of the articular layer is anatomically reduced with enough tendon tension (Figure 11C).

## DISCUSSION

The advantages of transtendon repair include the anatomically recovered footprint and preservation of the residual tendon.^10^ Superior biomechanical properties including smaller gap formation and superior failure strength are also presented.^11^ The reattached tendon can be returned to a near‐intact state following transtendon repair.^12^ Stuart et al.^13^ reported a 93% rate of good or excellent clinical outcomes and a 100% satisfaction rate in a 13‐year follow‐up of 15 patients. Castricini et al.^14^ described that among 33 patients, 93% had excellent clinical outcomes and no tear progression on magnetic resonance imaging at 33‐month follow‐up.

However, Liu et al.^15^ have raised concerns about the latent trauma on the residual tendon resulting from the use of the transtendon technique. Hirahara et al.^16^ considered that blindly introducing an anchor into the joint and creating a precise tendon hole are technically difficult. Zhou et al.^17^ affirmed that a healthy bursal layer could be damaged and tendon integrity could be destroyed by anchor insertion. Makki et al.^18^ believed that passing an anchor through the bursal layer resulted in iatrogenic injury. Yoon et al.^19^ described that a tendon of less than one‐half thickness could continue to degenerate under extraneous trauma. Yamakado et al.^20^ noticed that moderate histopathologic degeneration was present in over 90% of the macroscopically intact bursal layer. Andarawis et al.^21^ reported that PTRCTs altered the strain patterns of the residual tendon and led to tear enlargement. Yamanaka et al.^22^ described tear progression in 50% of PTRCTs and 28% developed to full‐thickness tendon tears within 12 months.

The ratio of the number of sutures crossing the tendon to the tendon volume is crucial for tendon repair.^23^ The tendon's durability relies on the number of sutures, but not the anchor used.^24^ Superior failure strength can be achieved from tendon repair using more sutures.^25^ The initial stability of the repaired tendon can be augmented by doubling the number of sutures.^26^ An ovine specimen study revealed that increasing the number of sutures remarkably lowered the risk of gap formation.^24^ Another ovine model study showed that increasing the number of anchors significantly increased the tendon failure strength due to the increased number of sutures.^27^

We consider that arthroscopic noninvasive transtendon repair possesses 4 advantages in the treatment of both‐sided PTRCTs. First, because a medial‐row anchor is not used, there is no threat of the anchor puncturing through the tendon, and the residual tendon can be thoroughly preserved without any trauma. Second, a tendon tear on the bursa can be effectively repaired while the articular layer can also be sufficiently restored. Third, 6 sets of DPSBs can offer superior failure strength, enough initial stability of the tendon, and decreased gap formation. Fourth, both the time and difficulty of the procedure are lowered, because a medial‐row anchor is not used and suture‐relay procedures are simplified.

Nevertheless, this technique still possesses some disadvantages: First, it is difficult to achieve anatomical reduction of an articular‐sided tendon tear because a medial‐row anchor is not used. Second, the spinal needle punctures the shoulder skin multiple times, which could potentially lead to joint infection. Third, this surgical technique demands high technical expertise from surgeons and has a long learning curve.

## DISCLOSURES

The authors (P.H., H.T., X.W., Y.F., Z.L., C.H.) declare that they have no known competing financial interests or personal relationships that could have appeared to influence the work reported in this article.
